# Ten actions to achieve gender equity among intensivists: the French Society of Intensive Care (FICS) model

**DOI:** 10.1186/s13613-022-01035-3

**Published:** 2022-07-02

**Authors:** Olfa Hamzaoui, Florence Boissier, Charlotte Salmon Gandonnière, Cécile Aubron, Laetitia Bodet-Contentin, Muriel Sarah Fartoukh, Mélanie Faure, Mercedes Jourdain, Julien Le Marec, Fabienne Tamion, Nicolas Terzi, Caroline Hauw-Berlemont, Nadia Aissaoui, FEMMIR Group for the SRLF Trial Group

**Affiliations:** 1 AP-HP, Service de réanimation polyvalente, Hôpital Antoine Béclère, Université Paris-Saclay, Clamart, France; 2 Médecine Intensive Réanimation, Hôpital Universitaire de Poitiers, INSERM CIC 1402 (ALIVE Group), Université de Poitiers, Poitiers, France; 3 Médecine Intensive Réanimation, INSERM CIC 1415, CRICS-TriGGERSep Network, CHRU de Tours, Tours, France; 4 Médecine Intensive Réanimation, Centre Hospitalier Régional Et Universitaire de Brest, Université de La Bretagne Occidentale, Brest, France; 5 Médecine Intensive Réanimation, INSERM CIC 1415, CRICS-TriGGERSep Network, CHRU de Tours and methodS in Patient-Centered Outcomes and Health ResEarch (SPHERE), INSERM UMR 1246, Université de Tours, Tours, France; 6 Service de Médecine Intensive Réanimation, Hôpital Tenon, APHP, and APHP, Sorbonne Université, Faculté de Médecine Sorbonne Université, Paris, France; 7 Diplôme d’études Spécialisées Médecine Intensive Réanimation, Nouzilly, France; 8 Médecine Intensive et Réanimation Membre de l’unité INSERM U1190 - Recherche Translationnelle Sur le Diabète, CHU de Lille, Lille, France; 9 AP-HP Sorbonne Université, Site Pitié-Salpêtrière Charles Foix, Service de Pneumologie, Médecine Intensive-Réanimation, Département R3S, INSERM, UMRS1158 Neurophysiologie Respiratoire Expérimentale Et Clinique, Sorbonne Université, Paris, France; 10 Médecine Intensive Et Réanimation, Hôpital Universitaire de Rouen, INSERM U1096 EnVi, Université Normandie, UNIROUEN, Rouen, France; 11 Inserm, U1042, CHU Grenoble Alpes, Medical Intensive Care Unit, Université Grenoble Alpes, 38000 Grenoble, France; 12 Médecine Intensive Réanimation, Hôpital Européen Georges Pompidou, AP-HP, Université de Paris, Paris, France; 13 Médecine Intensive Réanimation, APHP Centre, Cochin et Université de Paris, INSERM Unit 970, Cardiovascular Research Center (PARCC), Paris, France

**Keywords:** Gender equity, Working group, Sponsorship, Mentorship, Unconscius bias

## Abstract

In our recent survey, we aimed to collect information on perceived inequity as well as professional and personal fulfillment among women intensivists in France. For the 371 respondents out of the 732 persons who received the survey, the findings were unequivocal: for one-third of the respondents, being a woman was considered as an obstacle to careers or academic advancement, and for two thirds, pregnancy was viewed as a barrier to their career advancement. Gender discrimination had been experienced by 55% of the respondents. In 2019, to promote and achieve gender equity in the French Intensive Care Society (FICS), ten actions were initiated and are detailed in the present manuscript together with supporting data: (1) creation of a working group: the FEMMIR group; (2) promotion of mentorship; (3) implementation of concrete sponsorship; (4) transparency and public reporting of gender ratios in editorial boards; (5) workshops dedicated to unconscious gender bias; (6) workshops dedicated to improved women assertiveness; (7) role models; (8) creation of educational/information programs for young intensivists; (9) development of research on gender inequity and, as a perspective; and (10) development of a wide-ranging program. This review is aimed at providing a toolbox of organizational best practices designed to achieve gender equity. It is particularly important to share promising practical action engaged in our FEMMIR group with other concerned professionals around the world.

**Supplementary Information:**

The online version contains supplementary material available at 10.1186/s13613-022-01035-3.

## Introduction

Recently, the French College of Critical Care (CeMIR: Collège des Enseignants de Médecine Intensive Réanimation), reported that women accounted for 20–50% of the French critical care medicine workforce, depending on age and the geographic area, reaching 40% among under-40-year-old intensivists [1]. Nevertheless, and despite the fact that women have closed the sex gap with respect to their representation in the intensive care workforce, they remain underrepresented in the ranks of upper faculty. In 2020, only 8% of the professors in critical care medicine, 15% of the teachers, around 20% of the invited speakers in the French Intensive Care Society (FICS) congress and less than 30% of committee members in the FICS were women.

It is a well-established fact that women are underrepresented in leading positions, undervalued in their academic careers, and that they experience gender discrimination in scientific and health care disciplines throughout the world [2]. Accordingly, in our recent survey [3] including 371 respondents, gender discrimination had been experienced by 55% and one third of them declared that being a woman was an obstacle to careers and academic advancement [3]. Similar results were found in a previous international survey, where participants unanimously characterized critical care medicine as a specialty practiced predominantly by men [4]. Most of the women described experiences of being personally or professionally affected by gender inequity in their group [4]. Thematic analysis of the literature emphasized three key themes related to the advancement of women in medicine: (1) social barriers include maternal identity and cultural pressure with respect to work and family balance; (2) the importance of building resilience through role modeling, mentorship, and support from others and (3) practical difficulties including childcare needs, lack of timely career advice, and part-time working.

**Fig.1:**
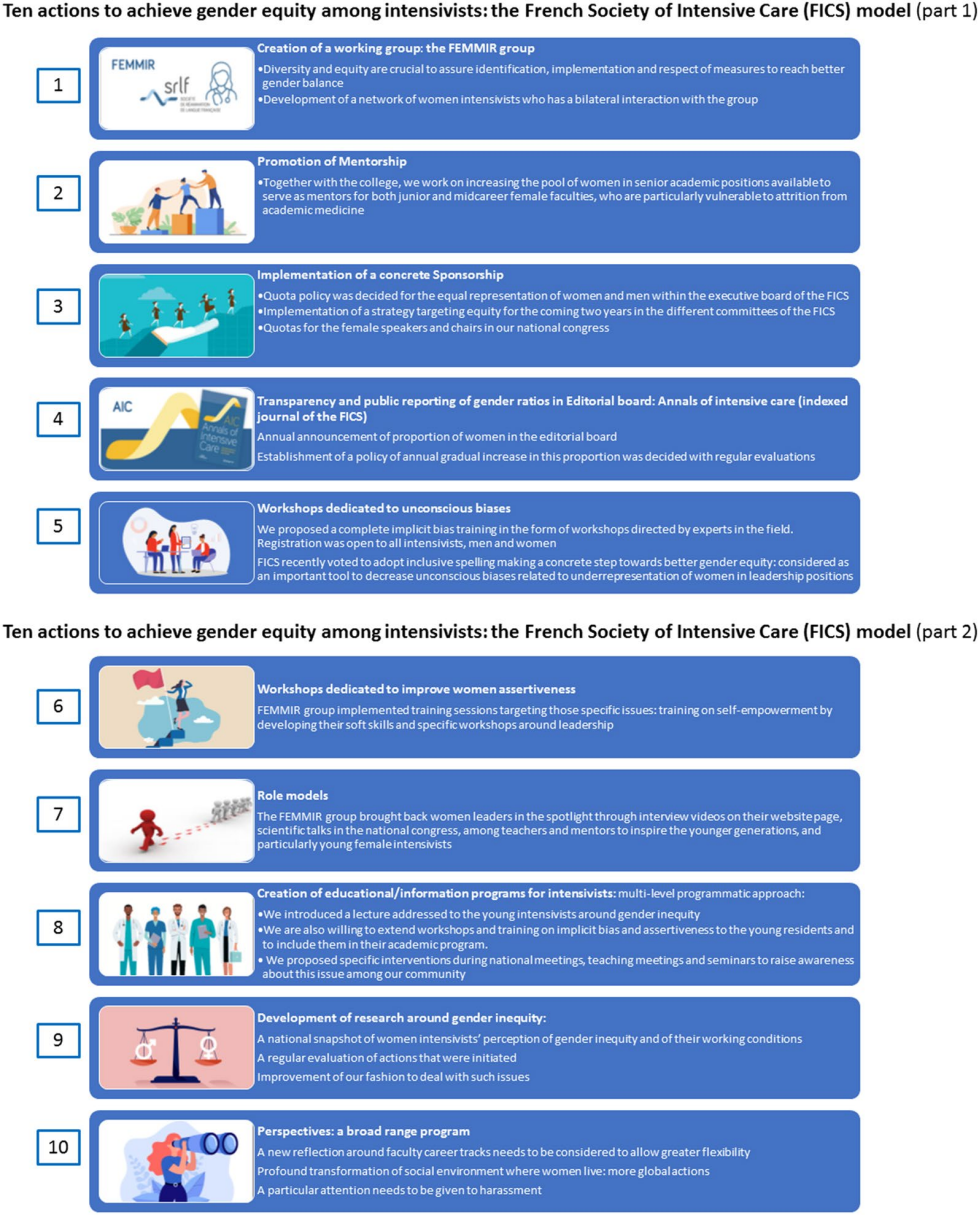
Ten actions to achieve gender equity among intensivists: the French Society of Intensive Care (FICS) model is the summary of the actions carried out by the FEMMIR group (Femmes Médecins en Médecine Intensive réanimation) during 2 years following o its creation. Part 1: represents actions 1 to 5, and Part 2 represents actions 6 to 10

In 2019, to promote and achieve gender equity, the FICS initiated several actions detailed below at the institutional level, the objective being to provide a toolbox [5] of organizational best practices designed to achieve gender equity (Fig. 1); that is also a key objective of the present review, in which we will highlight the best practices applicable to all specialties and with regard to different interlocutors: national scientific societies, medical boards, etc. We are convinced of the importance of sharing the promising practical actions engaged in the FEMMIR group with other concerned professionals around the world.

### Creation of a working group: the FEMMIR group

Working groups on diversity and equity are crucial to ensure identification, implementation and respect of measures designed to reach better gender balance. For this reason, a working group, the FEMMIR (Femmes Médecins en Médecine Intensive Réanimation), was created in the FICS, including 14 members (12 women and 2 men). Among the 10 women, two are invited members and not intensivists: one is the previous executive manager of the FICS, who worked for 30 years alongside numerous leaders, mainly men, and the second is an expert on gender equity and a professional coach in private companies. The working group rapidly developed a network of women intensivists for a two-way interaction; they relay initiatives, publicize the actions among the intensivist community at large and fuel by their suggestions the FEMMIR group actions.

Our group adopted a strategy based on the “listen, learn, and lead through actions’’ concept, with a view to taking practical actions to accelerate progress on gender equity in the scientific community and among intensivists. The current and the elected president of the FICS, the general secretaries and all the members of the executive committee are committed to enabling and promoting gender equity, which is considered as a major strategic opportunity and priority.

### Promotion of mentorship

Mentorship is associated with increased career satisfaction, faculty retention, research productivity and career advancement [6]. Women are less likely than their male colleagues to have a mentor through varying levels of training and are often less satisfied with their mentorship experiences [7–9]. In a recent study, Vranas et al. [10] demonstrated that female senior authors are significantly more likely than male senior authors to publish with female first and middle authors, suggesting that women may mentor and collaborate with other women more often than men do [10, 11]. These findings highlight the potential value of efforts to increase the pool of women in senior academic positions available to serve as mentors for both junior and midcareer female faculty, who are particularly vulnerable to attrition from academic medicine [7–9].

In this regard, together with the CeMIR, our group endeavors to increase the number of women among lecturers, by providing a link between academic institutions and the network of women that was created at the same time as the FEMMIR. In addition, this French women intensivists’ network aims to be more inclusive by recruiting more women among the researchers and involving them in different studies conceived by the FEMMIR members.

### Implementation of concrete sponsorship

Sponsors differ from mentors, as sponsors have both the position and the power to advocate publicly for the advancement of nascent talent within their organizations [12]. Sponsorship programs in the corporate world help raise women’s visibility, enhance their credibility, and advance them into upper levels of leadership [12, 13]. A number of studies have focused on the promotion gap; among them, a 2014 cross-sectional study [14] showed that gender disparity with respect to promotion remained, even after accounting for age, experience, specialty and research productivity. Similar results in 2018 reported that over 17 years, among 1273 faculty members at 24 U.S. medical schools, women were less likely than men to reach leadership positions, even after adjustment for publication-related productivity [15]. With this in mind, sponsorship may help to address the gender gap in leadership positions and scholarly activities within critical care. For example, between 2010 and 2016, women comprised only 5–31% of speakers at five major critical care conferences worldwide [16]. They have also been absent from the development of high-impact critical care consensus statements and clinical practice guidelines in recent years [17–19]. Despite their unpopularity among genders [20], quotas and targets have improved career advancement [21, 22], especially when supported by robust reporting [20].

Together with the executive committee of the FICS, the FEMMIR group has taken some concrete actions to sponsor women among intensivists and to increase their visibility: (1) recently, quota policy was decided on, in view of equal representation of women and men on the executive board of the FICS. (2) Quotas were also decided on for the female speakers and chairs in our national congress: to help the organizing committee, we established a list of women experts in each field based on their refereed publications in international peer review journals. (3) Implementation of a 2-year strategy targeting equity in the FICS committees: each year, some of the available positions will be reserved for female candidates, until 50% of the positions are held by women. We have carried out a promotion campaign on these available positions via our mailing lists and the social media. All of these actions were voted on either by all the members of the FICS or by the members of the executive committee.

### Transparency and public reporting of gender ratios in editorial boards

In various studies, the percentage of female editorial board members was consistently much lower than that of males [23–30]. Many reports have detailed, in addition to the gender inequities associated with medical journals [29, 30], barriers at this level that aggravate disparities in publishing, which in turn affect future grant funding, academic promotion and compensation, thereby slowing the advancement of women and preventing them from reaching their full potential capacities.

Greater participation of women on editorial boards may improve the quality and diversity of the review process, as suggested by a recent study on editorial board reviewer behavior that found significant differences in some aspects between men and women [31].

We are convinced that transparency is fundamental to achieve equity for underrepresented groups. Recommendations to promote transparency include issuing an annual equity report publishable on the website along with annual announcements on the proportion of women in published papers as first and/or senior authors. Accordingly, together with the executive committee of the FICS and the editor-in-chief of the peer reviewed and indexed journal of our national society (Annals of Intensive Care), we have decided on a public annual announcement of the proportion of women on the editorial board, which is presently 7 women among 41 editors. In addition, a policy of gradual annual increase of this proportion was decided, with regular evaluations.

To facilitate the membership of women on the editorial board of our journal, we proposed that more women be invited to prove themselves as journal reviewers, and that, if they perform successfully, they be considered for editorial board membership [32]. Although a quota system is not a definitive solution, it might be worthwhile used as a policy in the editorial boards. Indeed, with the help of the editor-in-chief, a range of expected number of women among editors has been established.

### Workshops dedicated to unconscious gender bias

Implicit gender biases that favor men do not necessarily arise from explicitly avowed beliefs [33, 34]. Unconscious bias refers to an implicit attitude, stereotype, motivation, or assumption that can occur without one’s knowledge, control, or intention. Forms of unconscious bias include gender bias, racial bias, and ageism [35]. Unconscious gender bias exists for many reasons [36]: men may be more assertive about seeking leadership roles, women may more commonly decline opportunities because of other professional priorities or caregiving responsibilities, leaders may habitually seek their customary colleagues and both men and women may implicitly associate science with males [37].

We are all aware that a major factor contributing to inequities is implicit bias, and that managing its effects requires an institutional commitment to the development of specific strategies. Accordingly, in a study by Girod et al. [33], the authors used a standardized, 20-min intervention to educate faculty about implicit biases and strategies for overcoming them. The assessment of the effect of faculty members’ perceptions of bias as well as their explicit and implicit attitudes toward gender and leadership indicated that the intervention significantly changed all of the faculty members’ perceptions of bias with regard to the eight measures predefined in the study [33].

Based on reports, in previous studies on the effectiveness of such measures, and to apply a more inclusive strategy among our different institutional leaders and search committees, we proposed complete implicit bias training in the form of workshops directed by experts in the field. Registration was open to all intensivists, men and women alike.

In the same way, the use of inclusive spelling in a language, such as French, where masculine nouns are used when both men and women are considered, can help to decrease unconscious biases related to underrepresentation of women in leadership positions. Consequently, the FICS recently voted to adopt inclusive spelling as a concrete step towards better gender equity.

### Workshops dedicated to improved women assertiveness

Women need to increase their confidence and belief in themselves—“Coaching” to help women recognize and overcome lack of self-esteem and lack of confidence in view of acceding to leadership positions may be useful. Therefore, we judged deemed it necessary to propose specific programs on individual and interpersonal levels: addressed behavior, knowledge, attitudes and skills of women faculty; they would also include mentoring, networking, training workshops, courses, and communication.

The FEMMIR group has organized training sessions on these specific issues: self-empowerment by developing soft skills and specific workshops on leadership. A targeted recruitment strategy was adopted by the executive committee of the FICS, with explicit solicitation of female applicants for leadership positions.

### Role models

The scarcity of women in leadership positions perpetuates inequity and is detrimental to trainees who are lacking in role models. Crucial interventions are required to increase the representation of women in leadership. A representative nationwide survey conducted in U.S. medical colleges showed that while female and male faculty members had similar leadership aspirations, women were less likely to have a sense of belonging and to perceive their institution as family-friendly and/or willing to make changes to address diversity goals [38]. The absence of women in higher ranks, especially as department chairs, may perpetuate the cycle. Women are underrepresented among residency program directors, who are role models, sponsors for career advancement [39] and on editorial boards of medical journals, which prioritize areas of research and determine which authors will have their work published [40].

The FEMMIR group has brought women leaders into the spotlight through interview videos on their website pages, scientific talks in national congresses, and among teachers and mentors liable to inspire the younger generations, and particularly young female intensivists.

### Creation of educational/information programs for young intensivists

In association with the CeMIR, the FEMMIR group has developed a complete program for intensive care residents aimed at decreasing gender-based discrimination and at appealing to, retaining and promoting women in our specialty. This has consisted in a multi-level programmatic approach aimed at more effectively, advancing the careers of women. As part of the teaching program, we introduced a lecture addressed to the young intensivists on gender inequity and its multiple causes in general and, more specifically, in intensive care. We are also planning to address workshops and training on implicit bias and assertiveness to the young residents and to have them included in their academic program. In addition, we have proposed specific interventions during FICS meetings to raise awareness on this issue in our community. Lastly, on our website (https://www.srlf.org/femmir), we have developed a pedagogical information tool on inequalities through creation of a specific tab including numerous scientific and socio-cultural studies.

### Development of research on gender inequity

For the FEMMIR group, development of research on the different aspects of gender inequity was deemed a priority for various reasons. The first reason was the need to have a national snapshot of women intensivists’ perceptions of gender inequity and their working conditions. Although there exist data from many countries throughout the world, there were no data available concerning French women physicians in general and French women intensivists in particular. This initial study [3] provided confirmation of previous worldwide data about obstacles that women may face during their professional careers. In addition, it represented a warning signal for the intensive care community and a stimulus for institutions to initiate needed changes. The second reason for such research is to regularly evaluate the actions having been initiated, as we are designing a study aimed at assessing the benefits and impact of FEMMIR on gender imbalance in FICS through comparison with other national and international societies. Finally, and as with any medical research, future studies, and taskforces will be necessary to improve our approach and management of such issues. In the very near future, the FEMMIR will be working with the FICS to improve intensivist well-being of by providing a taskforce.

### Perspectives: development of a wide-ranging program

In addition to programs focusing on individual factors, we are preparing future actions targeting a broad range of social factors and influences. Accordingly, several suggestions have emanated from our survey [3], including work–life balance and parental leave policies, provisions to stop the promotion clock, to increase and improve childcare resources and onsite lactation rooms [3, 41]. Most women physicians have children [3, 42] and are mothers, and they report having been discriminated against, because they were pregnant, took maternity leave or were breast-feeding [3, 43]. Future concrete actions must include an identified breastfeeding room in the FICS offices and in the national congress, as it is routine practice in other countries, while hoping that universities will follow the example.

Among the numerous potential causes of the sex gap in promotion, a disproportionate burden of family responsibilities, leading to difficulties in achieving work–life balance [3, 42], represents a major issue. With this in mind, critical perspectives have enabled researchers to question the underlying assumptions that produce and maintain social hierarchies, allowing them to imagine ways of transforming fields and practices in view of rendering them more equitable and inclusive. This will necessitate a profound transformation of women’s social environment, as it is one of the main factors likely to influence their careers.

## Strengths and limitations

To our knowledge, even though the above actions are frequently cited in the literature as bundles to follow [43], our work is largely unprecedented, insofar as it is designed to be implemented in not only a scientific national society, but also a medical specialty. It is one of the first concrete experiences having helped to develop a multilevel program in favor of gender equity. It presents the advantage of giving turnkey solutions to different communities, providing a toolbox that can be easily exported to other countries, other specialties and other professional communities’ facing similar problems.

Nevertheless, some limitations need to be addressed; for example, our actions have not included an equally necessary fight against harassment in the workplace. In our survey and in other studies, nearly one out of three women physicians and clinician–researchers indicated that they have experienced moral and/or sexual harassment in the workplace [3, 44, 45] and it appears to be more common in academic medical centers than in community or outpatient medical settings. Moreover, our actions cannot target the problem of disproportionate family responsibilities, the main cause for women to leave the field of medicine or forgo advancement as it is a more general and societal problem.

As regards the previously identified problems that may hinder women’s careers, we are aware that actions need to be developed at a higher level: the government, other influential associations, and local officials. This is why a communication plan concerning these actors needs to be developed, the objective being to establish effective discussion and to propose a model facilitating women’s careers (Additional file 1: Appendix S1).

## Conclusions

Our experience has shown that faculty development programmers should actively engage and motivate leaders to ensure gender equity and that these initiatives should be further institutionalized, based on evidence regarding what has, and what has not helped to achieve this objective. It is important to share promising practical action engaged by our FEMMIR group with others around the world. We must commit to ensuring that gender equity becomes an equal priority alongside research and innovation.

## Supplementary Information

**Additional file 1.** This a presentation leaflet of the working group FEMMIR (Femmes médecins en médecine intensive réanimation) including missions, actions and demands of the group.

## Data Availability

Not applicable.
