# Supplementary material for: Ten actions to achieve gender equity among intensivists: the French Society of Intensive Care (FICS) model
Source: Ann Intensive Care. 2022 Jul 2;12:59. doi: 10.1186/s13613-022-01035-3 (PMC9250559; doi:10.1186/s13613-022-01035-3)
Supplement: Supplementary file 1 — Additional file 1. This a presentation leaflet of the working group FEMMIR (Femmes médecins en médecine intensive réanimation) including missions, actions and demands of the group. [file 13613_2022_1035_MOESM1_ESM.pdf]

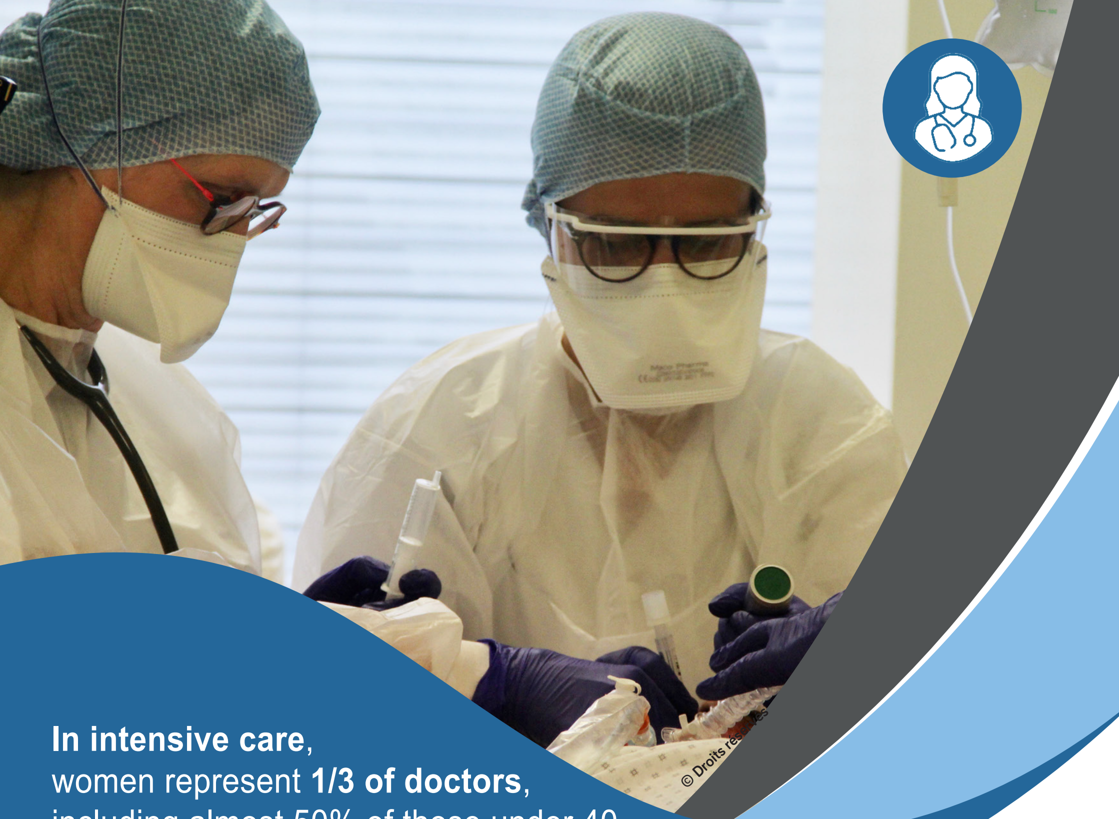

In intensive care,
women represent **1/3 of doctors**,
including almost 50% of those under 40.
Only 9% are professors or reach
an academic career.

FEMMIR

A working group for
Women doctors in Intensive care medicine

One group, 3 main objectives:
- improving working conditions
- reaching parity within FICS and intensive care medicine
- improving attractiveness of our specialty for young doctors

fics

TO HELP | TO HELP YOU | TO HELP EACH OTHER

OUR ACTIONS

to improve the representation of women in our medical specialty

PARITY WITHIN THE FICS

Amendment of rules of procedure and statutes of FICS, identification of positions for women and parity within the Executive Board.

VISIBILITY OF WOMEN

Networking to increase the visibility of women in the discipline among professors and speakers at our national conference.

SCIENTIFIC PUBLICATIONS

Research activities on gender in intensive care, two publications already published and several research projects in progress.

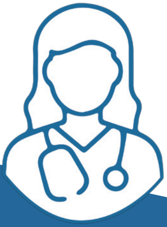

TRAINING SESSIONS / AWARENESS ACTIONS

Workshops on unconscious biases and on development of self-confidence.
Interventions to sensitize our entire community on gender inequalities.

OUR REQUESTS

Help us to make the difference by granting us:

A LISTENING TIME

A REAL INSTITUTIONAL COOPERATION

A FINANCIAL SUPPORT

Contact us
femmir@srlf.org

www.srlf.org/femmir
